# Seroprevalence of Major Viral Pathogens Associated with Bovine Respiratory Disease in Cattle in Türkiye and Scenario-Based Estimates of Economic Loss

**DOI:** 10.3390/vetsci13101117

**Published:** 2026-10-20

**Authors:** Pınar Ayvazoğlu Demir, Cemalettin Ayvazoğlu

**Affiliations:** 1 Department of Animal Health Economics and Management, Faculty of Veterinary Medicine, University of Kırıkkale, Kırıkkale 71450, Türkiye; 2 Department of Laboratory and Veterinary Health, Nihat Delibalta Göle Vocational School, Ardahan University, Ardahan 75700, Türkiye; cemalettinayvazoglu@ardahan.edu.tr

**Keywords:** BVDV, BHV-1, BRSV, PI-3, meta-analysis, Monte Carlo simulation, economic loss

## Abstract

Bovine Respiratory Disease (BRD) is a major health and economic concern in cattle production worldwide, resulting in substantial losses through reduced productivity, mortality, and treatment and veterinary service costs. This study aimed to estimate the seroprevalence of major BRD-associated viral pathogens in cattle in Türkiye and to quantify the potential economic loss per BRD-affected dairy cow using a scenario-based stochastic economic model. A systematic review and multilevel random-effects meta-analysis were conducted in accordance with the PRISMA 2020 guidelines. Thirty-one eligible studies published between 2000 and 2026 were included in the systematic review. Only antibody-based estimates obtained using enzyme-linked immunosorbent assay (ELISA) or serum neutralization (SN) tests were included in the quantitative seroprevalence meta-analysis, whereas PCR-based estimates were retained for descriptive evaluation. The pooled seroprevalence estimates were 72.0% (95% CI: 43.8–89.5) for bovine respiratory syncytial virus (BRSV), 59.5% (95% CI: 28.7–84.3) for parainfluenza virus type 3 (PI-3), 55.5% (95% CI: 42.1–68.2) for bovine viral diarrhea virus (BVDV), and 43.6% (95% CI: 15.7–76.1) for bovine herpesvirus type 1 (BHV-1). In the multivariable multilevel meta-regression, the overall effects of viral pathogen, diagnostic method, and population status on seroprevalence were not statistically significant (*p* > 0.05). Egger’s weighted regression tests did not indicate statistically significant funnel-plot asymmetry for any of the four pathogens. The potential economic loss per BRD-affected dairy cow was estimated using a Monte Carlo simulation model with 10,000 iterations. The mean potential economic loss was USD 331.63 per affected cow, with a median of USD 330.31 and a 95% uncertainty interval of USD 245.90–423.97. Live weight loss, treatment and veterinary service costs, mortality-related losses, and milk yield loss were the major components of the scenario-based economic loss. These findings indicate substantial exposure to BRD-associated viral pathogens in cattle populations in Türkiye and demonstrate that BRD may be associated with considerable economic consequences in affected dairy cattle. The results provide evidence to support BRD surveillance and control strategies and the prioritization of preventive measures. Future multicenter epidemiological studies using standardized methodologies and representative cattle populations are warranted to improve the precision and generalizability of national estimates.

## Simple Summary

Bovine respiratory disease (BRD) is a major cause of disease and economic loss in cattle. This study evaluated the prevalence and economic impact of four major BRD-associated viruses in Türkiye using data from 31 studies published between 2000 and 2026. The pooled seroprevalence estimates were 72.0% for BRSV, 59.5% for PI-3, 55.5% for BVDV, and 43.6% for BHV-1. Monte Carlo simulation estimated a mean potential economic loss of USD 331.63 per BRD-affected dairy cow. These findings indicate widespread exposure to BRD-associated viral pathogens in cattle populations in Türkiye and highlight the importance of surveillance and control strategies.

## 1. Introduction

Bovine respiratory disease (BRD) is a multifactorial disease complex resulting from interactions among infectious agents, host-related factors, and environmental conditions. Viral pathogens are important components of this complex because they can cause primary infections and increase susceptibility to secondary bacterial infections. The viral pathogens commonly associated with BRD include bovine respiratory syncytial virus (BRSV), bovine herpesvirus type 1 (BHV-1), bovine viral diarrhea virus (BVDV), and bovine parainfluenza-3 virus (PI-3) [1].

Among these viral pathogens, BVDV is one of the most extensively studied in terms of its economic consequences. BVDV is the causative agent of bovine viral diarrhea (BVD) and is associated with substantial economic losses in cattle production worldwide [2,3,4]. BVDV infection can result in reduced milk yield and live weight gain, calf losses, abortions, stillbirths, and impaired reproductive performance, as well as costs associated with veterinary services, diagnosis, treatment, and disease control programs [3,4]. Persistently infected (PI) animals play a central role in BVDV epidemiology because they continuously shed the virus and contribute to the persistence of infection within cattle herds [5,6].

The economic consequences associated with BVDV illustrate the importance of considering production losses in addition to mortality when assessing the potential economic impact of BRD-associated viral infections. Exposure to PI animals has been reported to be associated with reduced average daily gain and feed efficiency, as well as adverse effects on morbidity and mortality [7]. These findings indicate that the economic consequences associated with viral diseases may extend beyond mortality, with reductions in production efficiency potentially representing an important component of total economic loss. Therefore, comprehensive economic assessments should consider both direct disease- and control-related expenditures and production losses [3,4].

The epidemiological distribution of BRD-associated viral pathogens may vary according to herd characteristics, management practices, biosecurity, animal movements, vaccination status, geographical conditions, and diagnostic methods [1]. BVDV is endemic in cattle populations in many countries, and reported seroprevalence estimates vary considerably among populations [8]. Although numerous regional studies in Türkiye have investigated the seroprevalence of BVDV, BHV-1, BRSV, and PI-3 infections, previous meta-analytic studies have primarily focused on specific infections, particularly BVDV and infectious bovine rhinotracheitis (IBR) [9]. Therefore, an integrated assessment of the available epidemiological evidence on the major BRD-associated viral pathogens and the heterogeneity among studies is needed for cattle populations in Türkiye.

The aim of this study was to synthesize the available scientific evidence on the seroprevalence of major BRD-associated viral pathogens (BVDV, BHV-1, BRSV, and PI-3) in cattle in Türkiye using a systematic review and multilevel random-effects meta-analysis and to estimate the potential economic loss per BRD-affected dairy cow using a Monte Carlo-based stochastic economic model.

## 2. Materials and Methods

### 2.1. Study Design

This study was designed to determine the seroprevalence of major viral pathogens associated with bovine respiratory disease (BRD) complex in cattle in Türkiye and to estimate the potential economic loss per BRD-affected dairy cow using a multilevel random-effects meta-analysis and stochastic economic modeling based on Monte Carlo simulation.

### 2.2. Literature Review Strategy

**Figure 1:**
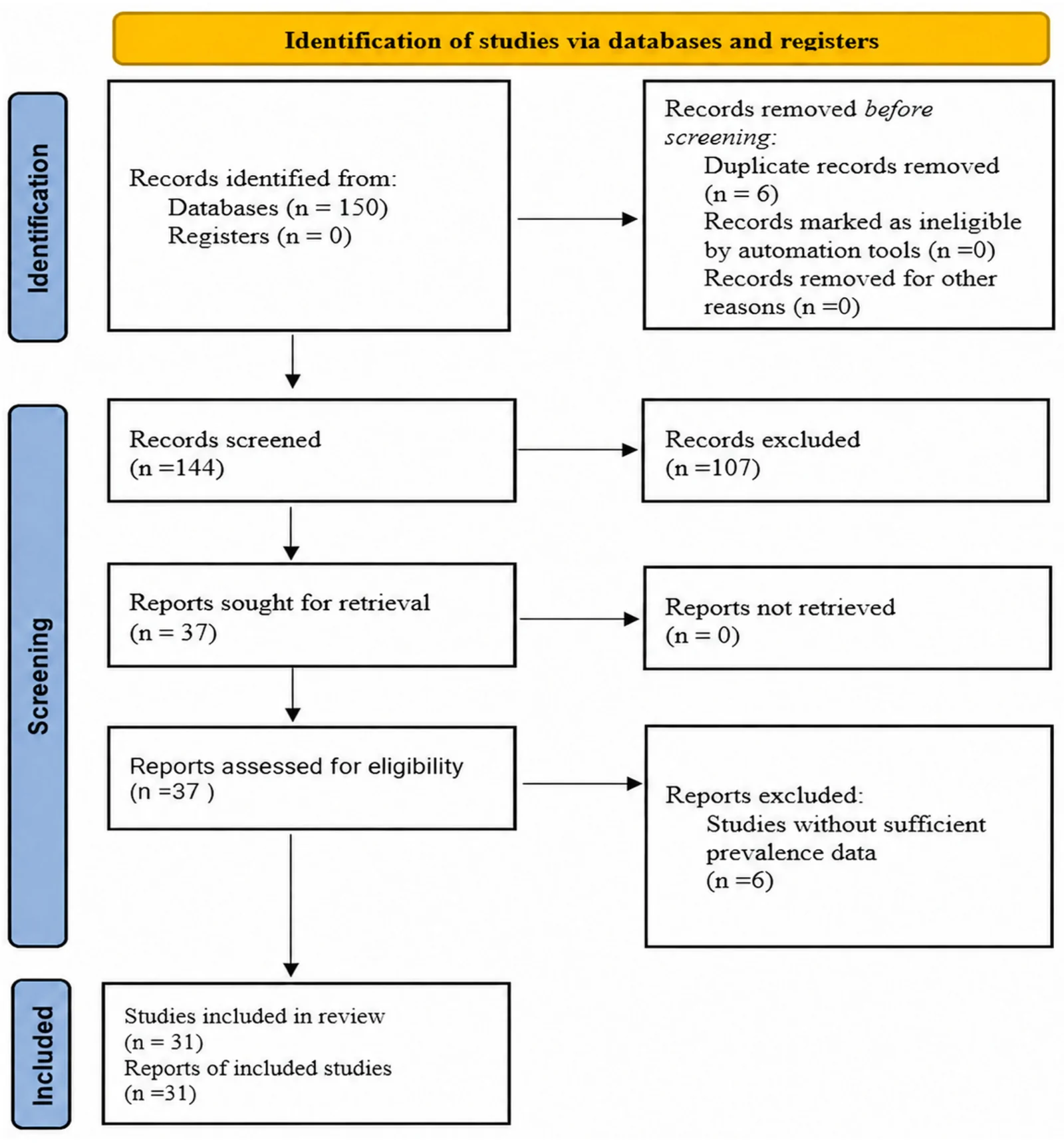
Flow diagram of literature search and selection.

The literature search was conducted using Google Scholar between 1 and 15 March 2026 and covered studies published from 1 January 2000 to 15 March 2026. The combined search expression was “cattle + prevalence + BVDV + BHV-1 + BRSV + PI-3 + Turkey”. The initial search yielded 150 records, which were screened according to the eligibility criteria described below. When the combined search expression was subsequently rechecked using AND and OR operators, the approximate number of results displayed by Google Scholar was also 150. Separate systematic searches were not conducted for each pathogen. This systematic review was conducted in accordance with the PRISMA 2020 (Preferred Reporting Items for Systematic Reviews and Meta-Analyses) guidelines [10]. A total of 150 records were identified through the literature search. After removal of duplicates, 144 records were screened based on their titles and abstracts. A total of 107 records were excluded, including studies involving non-bovine species, review articles, and studies that did not provide sufficient epidemiological data. The review protocol was not prospectively registered. The full texts of the remaining 37 studies were assessed for eligibility. Following full-text assessment, six studies that did not provide sufficient data to allow calculation of positivity rates were excluded, and a total of 31 studies were included in the study. The study selection process was conducted according to predefined inclusion and exclusion criteria and is presented in the PRISMA 2020 flow diagram (Figure 1).

### 2.3. Inclusion and Exclusion Criteria and Data Extraction

Original research studies reporting epidemiological data on the major viral pathogens associated with the Bovine Respiratory Disease (BRD) complex in cattle in Türkiye [Bovine Viral Diarrhea Virus (BVDV), Bovine Herpesvirus Type 1 (BHV-1), Bovine Respiratory Syncytial Virus (BRSV), and Parainfluenza Virus Type 3 (PI-3)] were included. The main inclusion criteria were that the studies were conducted in Türkiye, reported the sample size, and provided sufficient data to determine the number of positive animals and/or the positivity rate.

Review articles, case reports, conference abstracts, experimental infection studies, studies conducted in animal species other than cattle, duplicate publications, and studies lacking sufficient data to calculate the positivity rate were excluded.

For each study, the first author, publication year, study region, sample size, characteristics and health status of the animal population, vaccination status, viral pathogen investigated, diagnostic method used [enzyme-linked immunosorbent assay (ELISA), serum neutralization (SN) test, or polymerase chain reaction (PCR)], and reported positivity data were systematically recorded using a standardized data extraction form. Study selection, data extraction, and risk-of-bias assessment were performed independently by the two authors. Any disagreements were resolved through discussion and consensus. The extracted data were cross-checked for accuracy and consistency.

The methodological quality of the included studies was assessed using the Joanna Briggs Institute (JBI) Critical Appraisal Checklist for Studies Reporting Prevalence Data. Overall risk of bias was classified as low, moderate, or high based on the authors’ assessment of the nine JBI checklist items, considering the validity of prevalence estimates and the representativeness of the study populations. Detailed study-level risk-of-bias assessment results are presented in Supplementary Table S1.

### 2.4. Meta-Analysis

Antibody-positivity data obtained using enzyme-linked immunosorbent assay (ELISA) and serum neutralization (SN) tests were included in the meta-analysis, whereas PCR-derived results were excluded from the meta-analysis. PCR-based studies were evaluated descriptively as molecular detection findings.

Seroprevalence proportions were logit-transformed prior to analysis, and the standard errors of the logit-transformed proportions were calculated using the numbers of positive and negative animals. For interpretability, the results were back-transformed to the original proportion scale and presented as percentages [11].

A random-effects approach was adopted to account for the expected methodological and epidemiological differences among studies. Because individual studies could contribute multiple effect sizes for different viral pathogens, populations, or subgroups, a multilevel random-effects meta-analysis was applied to account for the dependence among effect sizes. Study-level random effects were incorporated into the model, and variance components were estimated using the restricted maximum likelihood (REML) method [12].

Pathogen-specific pooled seroprevalence estimates were calculated separately for BVDV, BHV-1, BRSV, and PI-3. For each estimate, 95% confidence intervals (CIs) and 95% prediction intervals (PIs) were calculated to reflect between-study variability. Study-level heterogeneity was expressed using the variance component (σ^2^) [13,14].

Multilevel random-effects meta-regression analyses were performed to evaluate differences in seroprevalence according to viral pathogen, population status (healthy/diseased), and serological diagnostic method (ELISA/SN). In addition, a multivariable multilevel random-effects meta-regression model including viral pathogen, population status, and diagnostic method simultaneously was fitted to account for potential confounding by pathogen type. Temporal trends in seroprevalence were evaluated by including publication year as a continuous moderator in a multilevel meta-regression model adjusted for viral pathogen. Vaccination status was not quantitatively evaluated because of the insufficient number of studies representing vaccinated populations.

Small-study effects were visually assessed using funnel plots generated separately for each viral pathogen, and funnel-plot asymmetry was examined using Egger’s weighted regression test [15]. A two-sided *p* < 0.05 was considered indicative of statistically significant funnel-plot asymmetry.

### 2.5. Economic Modeling

The potential economic losses in BRD-affected dairy cattle were estimated using a stochastic disease cost model based on Monte Carlo simulation. The target population of the model was defined as dairy cattle, and the economic analysis was designed to estimate the potential economic loss in a BRD-affected dairy cow. Seroprevalence estimates obtained from the meta-analysis were not included as inputs in the economic model.

The economic model was developed based on mortality ratio, treatment costs, veterinary service expenses, milk yield loss, and live weight loss parameters. To reflect the uncertainty in the model parameters, minimum, most likely, and maximum values were defined for each variable based on the literature and current market data, and triangular probability distributions were created using these values. The results were interpreted as scenario-based estimates of potential economic losses in BRD-affected dairy cattle.

The economic model included animal value loss because of mortality from viral infections associated with BRD, production losses resulting from milk yield loss and live weight loss, and disease management costs such as treatment expenses and veterinary service costs. Conversely, disease control methods such as vaccination programs, eradication practices, biosecurity measures, and other indirect operating costs are excluded from the model. Thus, the calculated economic losses are intended to reflect the potential economic losses in BRD-affected dairy cattle.

Treatment costs included medication, consumables, and application expenses, while veterinary service costs refer to examination, farm visits, and professional service fees. To avoid duplicate calculations between these two cost items, the expense categories have been clearly separated. Economic loss due to mortality was calculated by multiplying the estimated mortality ratio by the animal replacement value (USD/animal). To avoid simultaneously accounting for mortality-related and production losses in the same animals, milk yield and live weight losses were applied only to the surviving proportion of BRD-affected cattle (1 − *M*). Accordingly, economic loss due to milk yield reduction was calculated as (1 − *M*) × *M_loss_* × *P_milk_*, whereas economic loss due to live weight reduction was calculated as (1 − *M*) × *W_loss_* × *P_kg_*. For parameters partly based on scenario assumptions, the base-case value was anchored to the available literature, while the low and high values were defined as plausible bounds to represent uncertainty in the potential economic consequences of BRD. Because the available parameters were derived from studies conducted in different populations and disease contexts, they were not considered direct estimates for BRD-affected dairy cattle in Türkiye. Instead, they were used to define plausible parameter ranges for the scenario-based economic model. The parameter values used for the low, base, and high scenarios and their corresponding sources are presented in Table 1.

All monetary inputs used in the economic model were based on 2026 market values in Türkiye and expressed in US dollars (USD) using the average exchange rate for March 2026 (1 USD = 44.14 TRY). Studies published in earlier years were used only to determine biological and production-loss parameters, such as mortality, live weight loss, and milk yield loss.

The expected economic loss associated with BRD-related viral infections was estimated using the following equation:

*E*(*Loss*) = [(*C_treat_* + *C_vet_*) + (*M* × *V_animal_*) + (1 − *M*) × [(*M_loss_* × *P_milk_*) + (*W_loss_* × *P_kg_*)]

Here, *C_treat_* represents the cost of medication and treatment per animal, *C_vet_* represents the cost of veterinary practice, examinations, and farm visits, “*M*” represents the probability of mortality associated with BRD, *V_animal_* represents the replacement value of the lost animal, *M_loss_* represents the estimated milk loss per animal in kg/cow, *P_milk_* represents the unit milk price, *W_loss_* represents the estimated live weight loss per animal, and *P_kg_* represents the farm exit price per kilogram of live weight. The term (1 − *M*) represents the surviving proportion of BRD-affected cattle. The surviving proportion was applied to milk yield and live weight losses to prevent production losses from being calculated for animals already accounted for as mortality losses.

In the Monte Carlo simulation, 10,000 iterations were performed, and economic losses were calculated by taking random samples from the probability distributions defined for the parameters in each iteration. Based on the simulation results, the expected average economic loss per animal and its 95% uncertainty intervals were estimated.

### 2.6. Statistical Analysis

Meta-analyses and meta-regression analyses were performed using the MAJOR (Meta Analysis) module in jamovi (Version 2.7.17, The jamovi Project, Sydney, Australia), as described above. Economic analyses based on the Monte Carlo simulation model were performed using Microsoft Excel, and the expected mean economic loss per animal and its corresponding 95% uncertainty interval (UI) were estimated. Statistical significance was set at *p* < 0.05.

## 3. Results

A total of 31 studies deemed suitable for meta-analysis were evaluated in the study. These studies were published between 2005 and 2026 and conducted in different geographical regions of Türkiye. The included studies investigated the seroprevalence of major viral agents associated with Bovine Respiratory Disease (BRD), primarily Bovine Viral Diarrhea Virus (BVDV), Bovine Herpesvirus-1 (BHV-1), Bovine Respiratory Syncytial Virus (BRSV), and Parainfluenza-3 virus (PI-3). Information on sampling regions, diagnostic methods, population characteristics, and reported seroprevalence ratios for the studies is given in Table 2.

As shown in Table 2, the reported positivity rates varied considerably across regions and pathogens. ELISA and serum neutralization (SN) were the most commonly used serological methods, whereas PCR-based outcomes were excluded from the meta-analysis because they do not represent seroprevalence. In addition, vaccination status was not evaluated in the meta-analysis due to the insufficient number of studies. The 31 included studies comprised a total of 11,247 cattle, with individual study sample sizes ranging from 56 to 1288 animals. Following the exclusion of PCR-based outcomes, a total of 104 serological effect sizes were included in the quantitative meta-analysis. For BVDV, 4822 of 8998 animals tested were seropositive (53.6%); for BHV-1, 2035 of 6715 were seropositive (30.3%); for BRSV, 2198 of 3287 were seropositive (66.9%); and for PI-3, 2086 of 4059 were seropositive (51.4%).

**Figure 2:**
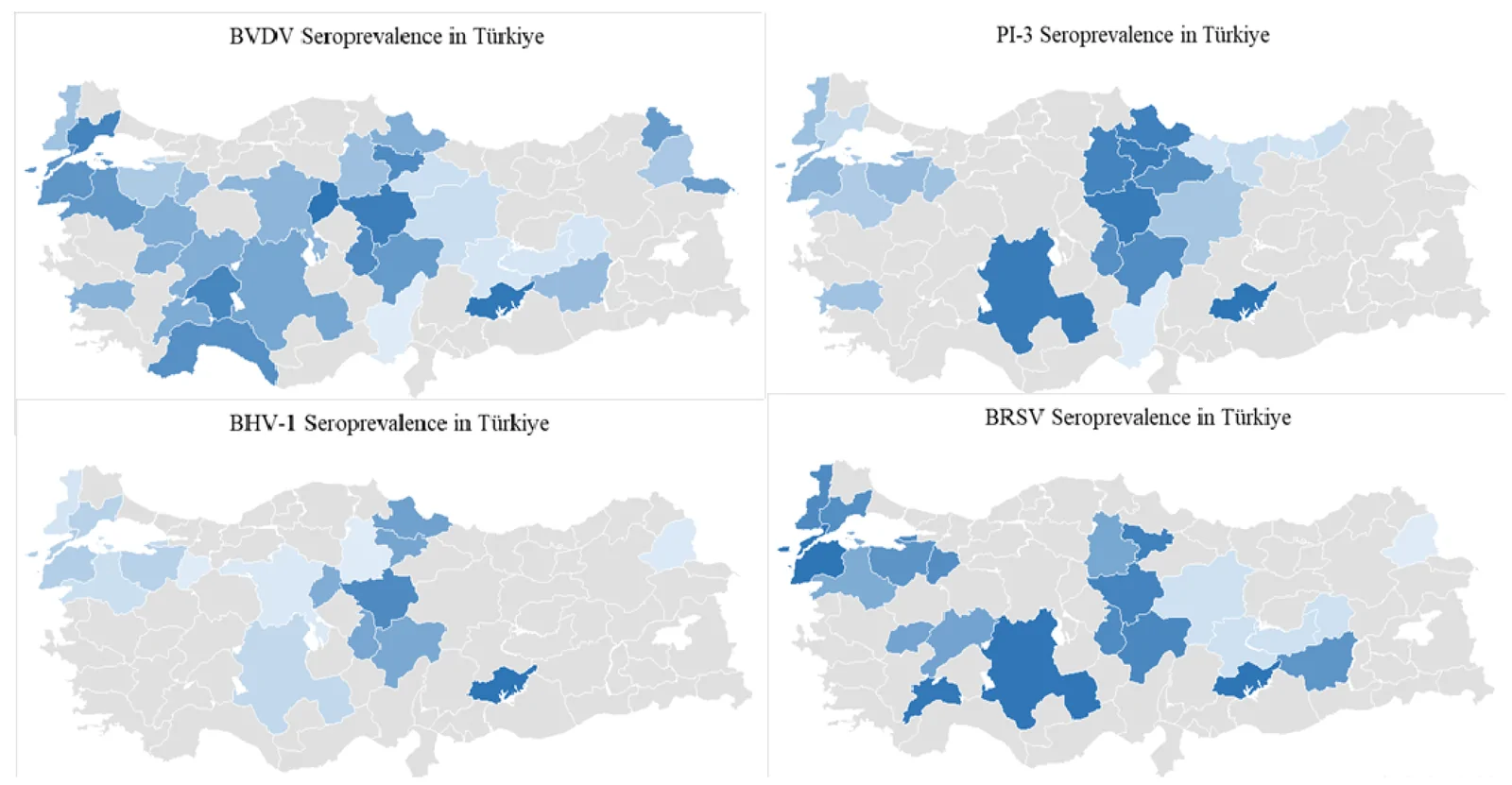
Geographical distribution of reported seroprevalence estimates for the major BRD-associated viral pathogens in Türkiye. Light, medium, and dark blue shades represent low (0–30%), moderate (30–60%), and high (>60%) seroprevalence levels, respectively. Gray areas indicate provinces with no available data.

The geographical distribution of the reported seroprevalence values of the major bovine respiratory viral pathogens in Türkiye is given in Figure 2. Considerable geographical variation was observed in the reported seroprevalence values across provinces in terms of BVDV, BRSV, BHV-1, and PI-3. The variation in seroprevalence levels among low (0–30%), medium (30–60%), and high (>60%) categories indicates regional exposure differences. Medium and high seroprevalence values were more frequently reported in the central and western regions of Türkiye, while suitable seroprevalence data were not found for some provinces in the Eastern Anatolia Region.

The pooled seroprevalence results of the major viral agents associated with BRD in cattle in Türkiye are presented in Table 3. The highest estimated pooled seroprevalence was observed for BRSV (72.0%; 95% CI: 43.8–89.5), whereas the lowest was observed for BHV-1 (43.6%; 95% CI: 15.7–76.1). The estimated pooled seroprevalence was 55.5% (95% CI: 42.1–68.2) for BVDV and 59.5% (95% CI: 28.7–84.3) for PI-3. However, the multilevel meta-regression analysis showed that the effect of pathogen type on seroprevalence was not statistically significant (F(3, 1.03) = 5.67, *p* = 0.290).

**Figure 3:**
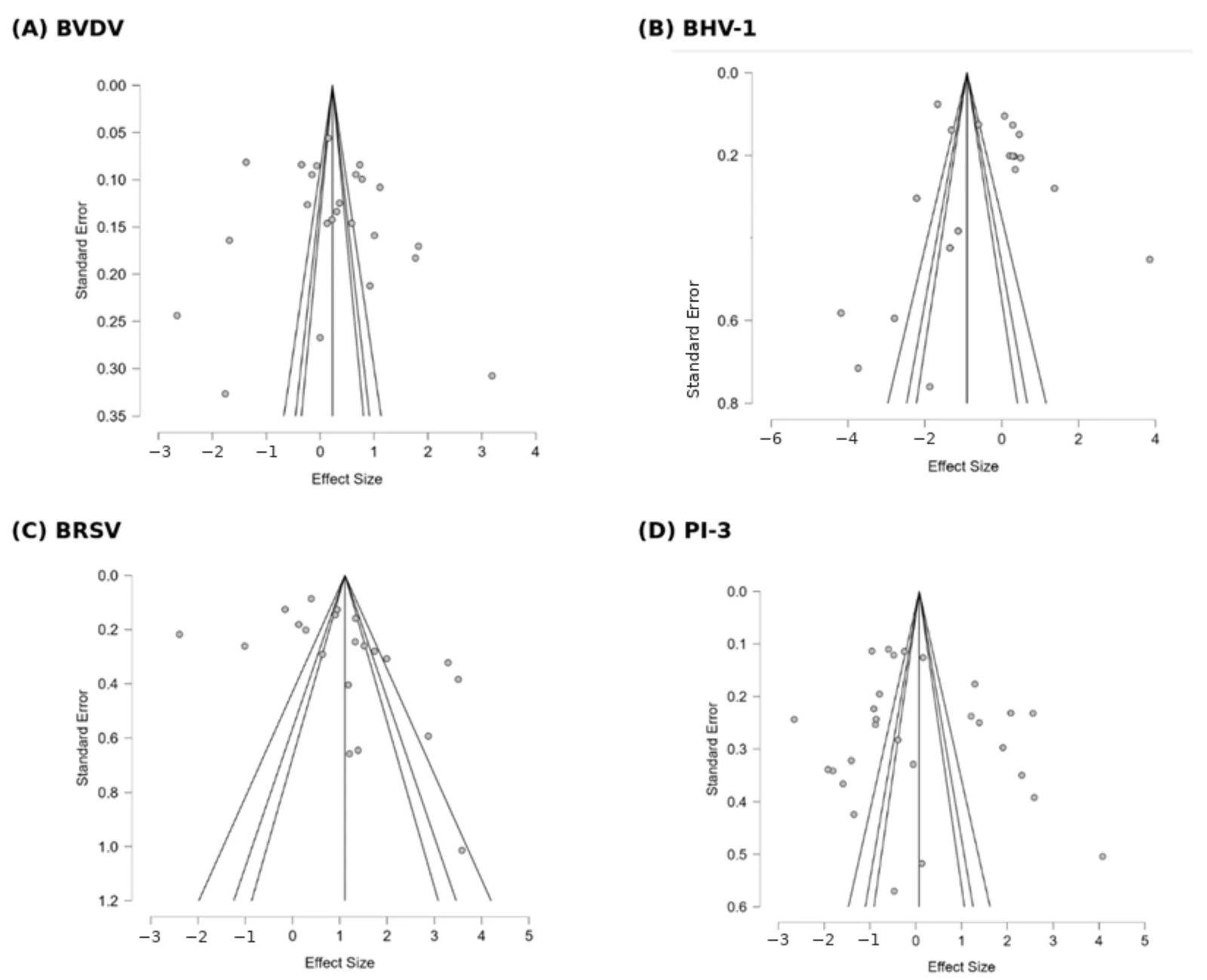
Funnel plots for the assessment of small-study effects across pathogen-specific meta-analyses: (**A**) BVDV, (**B**) BHV-1, (**C**) BRSV, and (**D**) PI-3.

The funnel plots for the four viral pathogens are presented in Figure 3. Egger’s weighted regression test did not indicate statistically significant funnel-plot asymmetry for BVDV (t(22) = 0.630, *p* = 0.535), BHV-1 (t(29) = 0.457, *p* = 0.651), BRSV (t(19) = 1.657, *p* = 0.114), or PI-3 (t(26) = 1.110, *p* = 0.277).

The results of the multilevel random-effects meta-regression according to population status are presented in Table 4. Although the estimated pooled seroprevalence was higher in diseased animals (64.6%; 95% CI: 31.5–87.8) than in healthy animals (44.1%; 95% CI: 28.6–60.9), after accounting for the clustering of multiple effect sizes within studies, the effect of population status on seroprevalence was not statistically significant (F(1, 6.58) = 2.56, *p* = 0.157).

The results of the multilevel random-effects meta-regression according to diagnostic method are presented in Table 5. The estimated pooled seroprevalence was 60.2% (95% CI: 34.8–81.0) in studies using ELISA and 43.4% (95% CI: 24.2–64.9) in studies using the serum neutralization (SN) test. Although the estimated seroprevalence was numerically higher in studies using ELISA, after accounting for the clustering of multiple effect sizes within studies, the effect of serological diagnostic method on seroprevalence was not statistically significant (F(1, 10.43) = 1.60, *p* = 0.234).

To control for the potential confounding effect of pathogen type, diagnostic method, viral pathogen, and population status were simultaneously included in a multivariable multilevel random-effects meta-regression model. The results of the analysis are presented in Table 6. The overall effects of diagnostic method [F(1, 2.541) = 3.735, *p* = 0.165], viral pathogen [F(3, 3.603) = 3.235, *p* = 0.156], and population status [F(1, 3.360) = 0.286, *p* = 0.626] on seroprevalence were not statistically significant.

In the analysis, publication year was included in the model to evaluate temporal trends in seroprevalence, while controlling for the effect of viral pathogen. No statistically significant association was observed between publication year and seroprevalence [F(1, 3.150) = 7.640, *p* = 0.066].

The Deterministic Scenario Analysis revealed that the potential economic loss per BRD-affected dairy cow was USD 115.02 in the low scenario, USD 288.23 in the base scenario, and USD 642.35 in the high scenario. According to the Monte Carlo simulation model, the mean potential economic loss per BRD-affected dairy cow was estimated at USD 331.63 (Table 7). The median economic loss was USD 330.31 per affected cow, with a 95% uncertainty interval of USD 245.90–423.97 per affected cow (Table 7).

## 4. Discussion

The present meta-analysis identified high pooled seroprevalence estimates for several major viral pathogens associated with BRD in Türkiye. The pooled seroprevalence estimates were 72.0% for BRSV, 59.5% for PI-3, 55.5% for BVDV, and 43.6% for BHV-1. In the multivariable multilevel meta-regression, the overall effect of viral pathogen on seroprevalence was not statistically significant after accounting for diagnostic method and population status (*p* = 0.156). Serological studies conducted in Türkiye also reveal that multiple seropositivity against agents such as BVDV, BHV-1, BRSV, and PI-3 is common, and that these infections often circulate together rather than individually. A study conducted in the Marmara Region reported that BRSV (73.0%), PI-3 (43.0%), and BVDV (41.4%) infections were common, and seroprevalence was particularly higher in large-scale dairy farms [51]. A study conducted in the Northeastern Anatolia Region also reported high seropositivity ratios for BVDV (58.9%), BHV-1 (61.5%), PI-3 (55.8%), BAV-1 (57.4%), and BAV-3 (50.2%), with only 2.3% of animals being seronegative for all agents and antibodies against two or more viral agents found in the majority of samples [52]. Similarly, in a study covering Samsun, Tokat, and Sivas provinces, it was reported that 74.4% of the sampled cattle were seropositive for at least one viral agent, and 30.7% carried antibodies against at least two or more viral agents [49].

The pathogen-specific seroprevalence estimates obtained for Türkiye varied across pathogens when compared with findings reported in previous meta-analyses. For BVDV, the pooled seroprevalence estimated in the present study (55.5%) was higher than the global antibody-based estimates of 42.7% (95% CI: 37.01–48.63%) reported by Su et al. [53] and 43% reported by Werid et al. [11]; however, it was very close to the pooled BVDV seroprevalence of 57.0% (95% CI: 44.4–69.5%) reported by Ran et al. [54] for dairy cattle in China. For PI-3, the pooled seroprevalence estimated in Türkiye (59.5%) was very similar to the pooled antibody-based seroprevalence of 60% (95% CI: 47–71%) reported by Werid et al. [55] for the general cattle population in a meta-analysis including 71 studies from 32 countries. This finding indicates that serological exposure to PI-3 in Türkiye is generally consistent with the prevalence reported globally. Similarly, the pooled BRSV seroprevalence estimated in the present study (72.0%) was higher than the pooled antibody-based seroprevalence of 63% (95% CI: 53–72%) reported in the global meta-analysis by Werid et al. [56], although it remained within the upper limit of the reported confidence interval. The pooled BHV-1 seroprevalence of 43.6% was also consistent with the estimates reported in the recent meta-analysis conducted in Türkiye by Arıkan et al. [57], particularly with the estimate obtained for large-scale studies. Overall, these findings indicate that serological exposure to the major viral pathogens associated with BRD is widespread in Türkiye and that the seroprevalence estimates obtained in the present study are generally consistent with the international literature.

The multilevel meta-regression analysis showed that, although seroprevalence was numerically higher in diseased cattle and in studies using ELISA, the differences were not statistically significant according to population health status (*p* = 0.157) or serological diagnostic method (*p* = 0.234). In the multivariable multilevel meta-regression, in which viral pathogen, diagnostic method, and population status were simultaneously included, none of these moderators showed a statistically significant overall effect on seroprevalence (all *p* > 0.05). In addition, no statistically significant association was observed between publication year and seroprevalence after controlling for viral pathogen (*p* = 0.066). Nevertheless, the non-significant meta-regression findings should not be interpreted as evidence of an absence of associations, given the substantial between-study heterogeneity, wide confidence and prediction intervals, and limited number of study clusters for some comparisons.

Because serological and molecular methods reflect different aspects of infection, PCR results were not considered appropriate for pooling with seroprevalence data. Indeed, Aslan et al. [24] reported a BVDV seropositivity of 70.89% by ELISA in the same study population, whereas viral genome positivity was 3.55% by RT-PCR. Therefore, ELISA and SN results were pooled in the present meta-analysis, whereas PCR results were excluded from the seroprevalence analysis.

Seropositivity alone is not an indicator of active infection or clinical disease; however, it reflects previous exposure to the infectious agent and herd-level exposure. Nevertheless, high seroprevalence is considered an important epidemiological indicator of widespread circulation of viral agents within the herd and continued infection pressure [53]. Indeed, BVDV seroprevalence ranging from 14.7% to 97.5% across studies, together with the wide 95% prediction intervals obtained for all four viral pathogens, indicates substantial between-study variability. This variability may be associated with differences in epidemiological conditions, herd characteristics, geographical regions, and sampling strategies. In parallel with this result, Ran et al. [54] and Zirra-Shallangwa et al. [58] reported that the combined evaluation of studies from different countries and production systems leads to significant heterogeneity in prevalence estimates and that epidemiological conditions are decisive in determining prevalence.

The presence of multiple viral agents circulating in the same herd increases the clinical severity of the disease and the associated economic losses because of the multifactorial nature of BRD. For this reason, the viral agents most frequently associated with BRD (BVDV, BHV-1, BRSV, and PI-3) were included in the current meta-analysis. It has been reported that BVDV and BHV-1 infections, which exhibit immunosuppressive properties, predispose to secondary bacterial infections, increasing the severity of the disease and the need for treatment, as well as causing decreased live weight gain, impaired feed utilization efficiency, reduced carcass quality, and decreased herd performance [42]. Also, it was reported that the economic impact of BVDV infections is not limited solely to direct losses because of clinical disease and mortality, but that indirect production losses such as reduced milk yield, growth retardation, impaired feed utilization efficiency, reproductive performance losses, and increased herd management costs also constitute significant components of the total economic burden [1,3,53]. Consistent with these results, the Monte Carlo-based economic modeling indicated that the potential economic losses in BRD-affected dairy cattle involve multiple cost components, including production losses and treatment and veterinary service expenses.

Hessman et al. [7] reported that exposure to persistently infected (PI) BVDV-positive cattle resulted in economic losses of USD 41.84–93.52 per animal in feedlots, largely due to reduced feed efficiency and growth performance. In the present study, the Monte Carlo simulation estimated a mean potential economic loss of USD 331.63 per BRD-affected dairy cow. The higher estimate obtained in the present model should be interpreted in light of differences in production systems, animal categories, and included cost components. Specifically, the current model incorporates treatment and veterinary service costs, mortality-related losses, milk yield loss, and live weight loss, whereas Hessman et al. [7] evaluated the economic consequences of exposure to PI cattle in a feedlot setting.

The economic effects of viral infections associated with BRD are not limited to mortality and treatment costs, but also include reduced milk yield and other production performance losses [3]. Köse et al. [59] reported that milk yield in the first lactation was significantly reduced in calves that experienced BRD and/or diarrhea in the neonatal period, and that there was a significant negative correlation between neonatal disease history and milk yield (*p* < 0.001; β = −2.144). Also, a reduction of approximately 20–30% in milk yield because of BVDV infection and respiratory system diseases is reported [16,58]. In the study, the 50–250 kg/cow milk yield loss scenario employed in the economic model was created by considering the average milk yield loss of 121.2 kg reported by Buczinski et al. [18]. In the present study, Monte Carlo simulation estimated a mean potential economic loss associated with milk yield loss of USD 74.27 per BRD-affected dairy cow (95% uncertainty interval: USD 36.48–120.35).

Economic model parameters for live weight loss (5–11,3–30 kg/animal) were developed based on field data reported by Demir and Bozukluhan [16]. Monte Carlo simulation estimated a mean potential economic loss associated with live weight loss of USD 86.40 per BRD-affected dairy cow. Similar to this, O’Donoghue et al. [60] reported a carcass weight loss of approximately 16 kg in subclinical cases and 24.1 kg in clinical cases. These two studies are important because they show that live weight loss affects the production performance of animals significantly and negatively.

The treatment cost range used in the economic model (USD 19.5–51.3 per animal) was defined by the authors based on 2026 treatment costs in Türkiye to represent uncertainty across low, base, and high scenarios. Monte Carlo simulation estimated a mean treatment and veterinary service cost of USD 88.90 per BRD-affected dairy cow. Similarly, O’Donoghue et al. [60] reported direct drug costs of approximately AUD 11.0, labor costs of AUD 15.0, and vaccination costs of AUD 7.67 in BRD cases. These findings indicate that the economic impact of BRD-associated viral infections cannot be explained solely by treatment costs and that veterinary services and production losses may also contribute substantially to the overall economic burden.

Mortality-related losses represented another substantial component of the model, with a mean estimated loss of USD 82.06 per BRD-affected dairy cow (95% uncertainty interval: USD 38.87–127.65). This component reflects the combined uncertainty in the assumed BRD-associated mortality ratio and animal replacement value.

The mean potential economic loss estimated in the present study was USD 331.63 per BRD-affected dairy cow. This estimate is higher than the range of USD 25–160 per animal reported by Houe [3] for BVDV infection, but remains below the approximately USD 410 per animal reported for severe clinical outbreaks. Overton [61] estimated the economic cost of BRD at USD 252–282 per case in dairy replacement heifers. The higher estimate obtained in the present study may be attributable to the combined consideration of mortality and replacement value, milk yield and live weight losses, and treatment and veterinary service costs in dairy cattle. Similarly, the economic loss calculated in the present study is consistent with the economic loss estimates reported by Stott et al. [62], who estimated that a single persistently infected (PI) cow could cause an economic loss of approximately £431 to the farm, and the range of £0–552 per cow reported by Su et al. [53]. This wide range of economic losses reported in the literature stems from differences in the clinical severity of the infection, the production system, the infection pressure, epidemiological conditions, the economic modeling approach, and the components included in the cost calculations. Indeed, Zirra-Shallangwa et al. [58] reported an economic loss of approximately 88 USD/animal because of BVDV infection in beef cattle, while Hessman et al. [7] reported a loss of 41.84–93.52 USD/animal in beef cattle exposed to persistently infected (PI) animals.

Arıkan et al. [57] evaluated the economic impact of BoAHV-1 infection in Türkiye and reported that the economic loss per infected animal ranged from approximately USD 285.18 to 383.21. In that study, economic losses were assessed particularly in relation to impaired reproductive performance, including age at first calving, calving interval, and abortion, whereas the present study considered production losses associated with respiratory disease, together with mortality, treatment, and veterinary service costs in dairy cows affected by BRD. Despite these differences in modelling scope, the mean economic loss estimated in the present study (USD 331.63 per affected cow) falls within the range reported by Arıkan et al. [57].

In his study evaluating the epidemiological and economic impacts of BVDV infections, Houe [63] reported that the annual economic loss from low virulence strains reached approximately $20 million USD/million calving, while that from high virulence strains reached approximately $57 million USD/million calving, and that an increase in infection incidence significantly increased economic losses. Similarly, Sacco et al. [64] reported that Bovine Respiratory Syncytial Virus (BRSV) causes approximately $1 billion USD in annual economic losses on a global scale because of mortality, decreased production performance, and treatment and vaccination costs. These findings indicate that the economic consequences of BRD-associated viral infections may extend beyond production losses at the individual-animal level and may have substantial economic implications for the livestock sector more broadly. Wang et al. [65] considered mortality, treatment costs, and live weight loss as important components of the economic burden of BRD, while Overton [61] reported that BRD in dairy replacement heifers may also contribute to longer-term economic losses through reduced growth, culling, and changes in future milk production. Differences in the economic loss components considered, animal populations and modeling approaches across studies may contribute to variation in BRD-related economic loss estimates. Furthermore, herd-level factors such as the introduction of new animals, herd size, cattle density, and contact with neighboring herds have been reported to be associated with the risk of BoHV-1 introduction [66]. These findings indicate that herd and management characteristics, which were not adequately reported across the included studies, should also be considered when interpreting the substantial between-study heterogeneity observed in the present meta-analysis.

The economic effects of BVDV infections are not limited to direct losses due to clinical disease and mortality but also include subclinical losses that negatively affect herd performance. Infected herds have been reported to experience adverse effects such as increased somatic cell counts, higher ratios of premature reformation, increased calf mortality, delayed age at first calving, and longer calving intervals [1,67,68]. In addition, abortions and reproductive losses, particularly in persistently infected (PI) animals, further increase the overall economic burden of the infection at the farm level [62].

Although the high seroprevalence found in this study does not directly prove the presence of persistently infected (PI) animals, it suggests that BVDV is widespread in herds and that PI animals may be present. In BVDV epidemiology, PI animals constitute the primary reservoir for the continuity of infection within the herd because of their continuous shedding of the virus throughout their lives, and are considered one of the most important epidemiological factors determining the success of control and eradication programs [3,63,69]. For this reason, identifying and removing PI animals from herds with high seroprevalence using antigen or molecular methods is among the fundamental control strategies for breaking the infection chain and reducing long-term economic losses [3,69]. Also, the reporting of high seropositivity ratios in unvaccinated herds supports the idea that viral agents circulate widely through natural infections in field conditions. In this context, Yeşilbağ and Güngör [51] reported that BRSV seropositivity reached 73.0% in unvaccinated cattle in the Marmara Region, and that high seropositivity ratios were also found against other BRD-related viral agents.

Economic studies evaluating BVDV control and eradication programs show that investments in biosecurity and preventive practices yield significant long-term economic gains [4]. In Norway, the national BVDV control program reportedly generated approximately €14 million in net present value [70]; in Ireland, the eradication program generated economic benefits approximately 14 times its costs [71]; and in Switzerland, the total economic benefit reached approximately twice the program cost [72]. Similarly, economic modeling studies conducted in the United Kingdom and Scotland reported that long-term BVDV control programs are economically sustainable [73,74]. At the farm level, vaccination programs with 80% effectiveness have been reported to provide net economic benefits [75]. Also, a meta-analysis by Theurer et al. [14] showed that the risk of BRD morbidity was reduced by 56% in vaccinated herds (RR = 0.44; 95% CI: 0.26–0.74). Vaccination status is important when interpreting antibody-based seroprevalence results, as serologically detected antibodies may reflect not only natural infection but also vaccine-induced immune responses [27]. However, vaccination status was not consistently reported across the studies included in the present meta-analysis, and the limited number of studies representing vaccinated populations precluded a separate quantitative analysis according to vaccination status. In addition, the literature search was conducted exclusively through Google Scholar using a combined search expression, which may have limited the identification of some pathogen-specific studies.

Furthermore, the most frequent methodological limitations of the included studies were related to sample selection and the representativeness of the study populations. A substantial proportion of studies were conducted on clinical cases, selected herds, or individual farms, potentially limiting the generalizability of the findings to the broader cattle population in Türkiye. Accordingly, the pooled seroprevalence estimates should be interpreted as indicators of viral exposure within the populations represented by the included studies, rather than as direct estimates of clinical BRD incidence or nationally representative seroprevalence.

## 5. Conclusions

In this study, the seroprevalence of major viral pathogens associated with BRD in Türkiye was systematically evaluated, and the findings demonstrated that exposure to BRD-associated viral pathogens is widespread among cattle populations in Türkiye. Among the viral pathogens examined, BRSV had the numerically highest pooled seroprevalence; however, differences in seroprevalence among pathogen types were not statistically significant. The economic modeling conducted in this study showed that potential economic losses in BRD-affected dairy cattle comprise multiple cost components, including treatment and veterinary service costs, mortality-related losses, milk yield loss, and live weight loss. Monte Carlo simulation estimated a mean potential economic loss of USD 331.63 per BRD-affected dairy cow.

In conclusion, this study represents one of the comprehensive investigations in Türkiye to jointly evaluate the pooled seroprevalence of major BRD-associated viral pathogens and the potential economic losses in BRD-affected dairy cattle. The findings may provide a scientific basis for developing BRD surveillance and control strategies, prioritizing resources, and planning cost-effective preventive programs.

## Figures and Tables

**Table 1:** Parameters used in the Monte Carlo simulation model for estimating potential economic losses in BRD-affected dairy cattle.

Parameters	Low Scenario	Base Scenario	High Scenario	Sources
Mortality ratio (%)	1	3	5	Based on Hessman et al. [7] and authors’ scenario assumptions
Live weight loss (kg/cow)	5	11.3	30	Demir and Bozukluhan [16]
Treatment cost (USD/cow)	19.5	30.8	51.3	Authors’ scenario assumptions based on 2026 Turkish treatment costs
Veterinary service cost (USD/cow)	25	50	90	Ankara Veterinary Medical Association, 2026 [17]
Milk yield loss (kg/cow)	50	120	250	Adapted from Buczinski et al. [18] with authors’ scenario assumptions
Milk price (USD/kg)	0.48	0.54	0.62	USK, 2026 [19]
Live weight price (USD/kg)	4.8	5.8	6.8	TUİK 2026 [20]
Animal replacement value (USD/cow)	2300	2700	3200	TIGEM, 2026 [21]

**Table 2:** Characteristics of the studies included in the systematic review of BRD-associated viral pathogens in cattle in Türkiye.

Study	Province(s)	Pathogen(s)	Diagnostic Method	Population Status	Vaccination Status	Sample Size (*n*)	Reported Positivity (%)
Akar [22]	Diyarbakır	BVDV	SN	Healthy	NR	281	15.7
Alpay et al. [23]	Gökçeada	BVDV BHV-1 BRSV PI-3	SN	Healthy	NR	75	14.7–58.7
Aslan et al. [24]	Kırıkkale Yozgat Çorum Ankara	BVDV BHV-1	ELISA	Diseased	Vaccinated	615	0.0–97.5
Avcı and Yavru [25]	Konya	BVDV	ELISA	Diseased	Unvaccinated	450	46.2
Avcı et al. [26]	Amasya Çorum, Yozgat Kayseri Nevşehir	BVDV BHV-1 BRSV PI-3	ELISA	Diseased	Unvaccinated	500	55.0–93.0
Aydın et al. [27]	Edirne	BRSV	ELISA	Healthy	Mixed	240	79.2
Baldan Toker [28]	Bursa	BVDV	PCR	Diseased	NR	193	2.6
Bilgili and Mamak [29]	Isparta	BVDV	ELISA	Healthy	Unvaccinated	460	75.2
Bulut et al. 2006 [30]	Konya	BVDV BHV-1	ELISA	Healthy	NR	1288	15.8–53.8
Demirsoy and Mamak [31]	Antalya	BVDV	ELISA	Diseased	Unvaccinated	470	68.5
Duman et al. [32]	Konya	BVDV BHV-1 BRSV PI-3	ELISA	Diseased	Unvaccinated	278	35.3–96.4
Erkılıç et al. [33]	Kars	BVDV BHV-1 BRSV	PCR	Diseased	Unvaccinated	200	1.5–4.0
Erol et al. [34]	Aydın	PI-3	SN	Healthy	Unvaccinated	288	38.2
İnce and Ayaz [35]	Afyon, Kütahya Uşak	BVDV	ELISA	Healthy	Unvaccinated	552	48.4
İnce et al. [36]	Afyon Uşak	BRSV	ELISA	Healthy	NR	557	59.8
Kadiroğlu et al. [37]	Diyarbakır	BVDV, BRSV	SN	Healthy	Unvaccinated	229	57.6–71.2
Kale et al. [38]	Burdur	BVDV BRSV	ELISA	Diseased	Unvaccinated	56	50.0–94.6
Koç Akpınar and Çitil [39]	Samsun	BVDV	ELISA	Mixed	Unvaccinated	404	55.5–64.2
Küçük et al. [40]	Burdur	BRSV	PCR	Diseased	Unvaccinated	200	11.5
Okur Gümüşova et al. [41]	Samsun	BVDV BHV-1 PI-3	SN	Healthy	Unvaccinated	188	53.2–88.8
Öner and Yeşilbağ [42]	Adıyaman	BVDV BHV-1 BRSV PI-3	SN	Healthy	Unvaccinated	240	85.4–98.3
Özbek et al. [43]	Malatya Elazığ Diyarbakır Bingöl Sivas	BVDV	PCR	Diseased	NR	129	10.90
Paksoy et al. [44]	Konya Adana	BVDV BRSV PI-3	ELISA	Diseased	NR	274	6.6–8.4
Şimşek et al. [45]	Diyarbakır	BVDV	ELISA	Healthy	Unvaccinated	109	71.6
Tan et al. [46]	Aydın	BVDV	SN	NR	Unvaccinated	288	86.0
Yavru et al. [47]	Konya	BVDV BHV-1 BRSV PI-3	SN	Healthy	Unvaccinated	254	44.1–57.1
Yavru et al. [48]	Konya	BVDV	ELISA	Healthy	Unvaccinated	202	73.3
Yazıcı et al. [49]	Samsun Tokat Sivas	BVDV PI-3	SN	Healthy	Unvaccinated	936	15.3–78.4
Yazıcı et al. [50]	Samsun Ordu Tokat Giresun Trabzon Rize	PI-3	SN	Healthy	Unvaccinated	442	12.8–29.6
Yeşilbağ ve Güngör [51]	Bursa Yalova Balıkesir Çanakkale Bilecik Edirne Tekirdağ	BVDV BHV-1 BRSV PI-3	SN	Healthy	Unvaccinated	584	0.0–97.3
Yıldırım et al. [52]	Ardahan Kars Iğdır	BVDV	ELISA	Healthy	Unvaccinated	265	56.3–60.7

BVDV, bovine viral diarrhea virus; BHV-1, bovine herpesvirus type 1; BRSV, bovine respiratory syncytial virus; PI-3, parainfluenza-3 virus; ELISA, enzyme-linked immunosorbent assay; SN, serum neutralization test; PCR, polymerase chain reaction; NR, not reported. Mixed: studies including both healthy and diseased cattle.

**Table 3:** Multilevel random-effects meta-analysis of seroprevalence of major BRD-associated viral pathogens in cattle in Türkiye.

Pathogens	Effect Sizes (k)	Study Clusters (*n*)	Pooled Seroprevalence (%)	95% CI (%)	95% PI (%)	Study-Level Variance (σ^2^)
BVDV	24	23	55.5	42.1–68.2	8.3–94.5	1.535
BHV-1	31	10	43.6	15.7–76.1	0.7–98.8	3.900
BRSV	21	11	72.0	43.8–89.5	4.1–99.4	3.101
PI-3	28	11	59.5	28.7–84.3	1.7–99.2	3.672

Pooled estimates were obtained using multilevel random-effects meta-analysis with effect sizes nested within studies. Only antibody-based estimates obtained by ELISA or serum neutralization (SN) were included; PCR-based estimates were excluded from the quantitative seroprevalence meta-analysis. Proportions were analyzed on the logit scale and back-transformed to percentages for presentation. CI, confidence interval; PI, prediction interval.

**Table 4:** Multilevel random-effects meta-regression of BRD-associated viral seroprevalence in cattle in Türkiye according to population status.

Population Group	Effect Sizes (k)	Pooled Seroprevalence (%)	95% CI	95% PI
Healthy	71	44.1	28.6–60.9	1.7–97.3
Diseased	33	64.6	31.5–87.8	1.5–99.6

The between-group difference was not statistically significant (F(1, 6.58) = 2.56, *p* = 0.157).

**Table 5:** Multilevel random-effects meta-regression of BRD-associated viral seroprevalence in cattle in Türkiye according to serological diagnostic method.

Diagnostic Methods	Effect Sizes (k)	Pooled Seroprevalence (%)	95% CI	95% PI
Serum Neutralization Test (SN)	59	43.4	24.2–64.9	1.0–98.3
ELISA	45	60.2	34.8–81.0	2.8–98.7

The between-group difference was not statistically significant (F(1, 10.43) = 1.60, *p* = 0.234).

**Table 6:** Multivariable multilevel meta-regression of BRD-associated viral seroprevalence in cattle in Türkiye.

Moderator	F	df1	df2	*p*
Diagnostic method	3.735	1	2.541	0.165
Viral pathogen	3.235	3	3.603	0.156
Population status	0.286	1	3.360	0.626

**Table 7:** Monte Carlo simulation results for the potential economic loss per BRD-affected dairy cow.

Economic Loss Components	Mean (USD/Cow)	Median (USD/Cow)	95% Uncertainty Interval (USD/Cow)
Treatment + veterinary service cost	88.90	87.87	61.80–118.42
Mortality-related loss	82.06	81.85	38.87–127.65
Milk yield loss	74.27	72.13	36.48–120.35
Live weight loss	86.40	82.26	38.87–150.94
Total economic loss	331.63	330.31	245.90–423.97

## Data Availability

The raw data supporting the conclusions of this article will be made available by the authors on request.

## Supplementary Materials

The following supporting information can be downloaded at: https://www.mdpi.com/article/10.3390/vetsci13101117/s1. Table S1. Risk-of-bias assessment of the included studies using the Joanna Briggs Institute (JBI) critical appraisal checklist.
