# Supplementary material for: Seroprevalence of Major Viral Pathogens Associated with Bovine Respiratory Disease in Cattle in Türkiye and Scenario-Based Estimates of Economic Loss
Source: Vet Sci. 2026 Oct 20;13(10):1117. doi: 10.3390/vetsci13101117 (PMC13709826; doi:10.3390/vetsci13101117)
Supplement: Supplementary file 1 [file vetsci-13-01117-s001.zip › vetsci-4577566-supplementary.pdf]

No	Study	Q1	Q2	Q3	Q4	Q5	Q6	Q7	Q8	Q9	Overall risk of bias
1	Akar, 2017	Yes	Unclear	Unclear	Yes	Unclear	Yes	Yes	Yes	Unclear	Moderate
2	Alpay et al., 2014	No	Yes	No	Yes	No	Yes	Yes	Yes	Unclear	High
3	Aslan et al., 2015	No	No	Yes	Yes	No	Yes	Yes	Yes	Unclear	High
4	Avcı et al., 2014	Yes	Yes	Yes	Yes	Yes	Yes	Yes	Yes	Unclear	Low
5	Avcı & Yavru, 2013	No	No	Yes	Yes	No	Yes	Yes	Yes	Yes	High
6	Aydın et al., 2024	No	Yes	Yes	Yes	No	Yes	Yes	Yes	Unclear	High
7	Baldan Toker, 2019	No	No	Unclear	Yes	No	Yes	Yes	Yes	Unclear	High
8	Bilgili & Mamak, 2019	Unclear	Unclear	Yes	Yes	Unclear	Yes	Yes	Yes	Unclear	Moderate
9	Bulut et al., 2006	Yes	Yes	Yes	Yes	Yes	Yes	Yes	Yes	Unclear	Low
10	Demirsoy & Mamak, 2020	Unclear	Unclear	Yes	Yes	Unclear	Yes	Yes	Yes	Unclear	Moderate
11	Duman et al., 2009	Yes	No	Yes	Yes	Yes	Yes	Yes	Yes	Yes	Moderate
12	Erkılıç et al., 2024	No	No	Yes	Yes	No	Yes	Yes	Yes	Yes	High
13	Erol et al., 2007	No	Unclear	Yes	Yes	No	Yes	Yes	Yes	Unclear	High
14	İnce et al., 2022	Yes	Yes	Yes	Yes	Yes	Yes	Yes	Yes	Unclear	Low
15	İnce & Ayaz, 2023	Yes	Yes	Yes	Yes	Yes	Yes	Yes	Yes	Unclear	Low
16	Kadiroğlu et al., 2020	Yes	Yes	Unclear	Yes	Yes	Yes	Yes	Yes	Unclear	Moderate
17	Kale et al., 2013	No	No	No	Yes	No	Yes	Yes	Yes	Yes	High
18	Koç Akpınar & Çitil, 2022	Unclear	Unclear	Yes	Yes	Yes	Yes	Yes	Yes	Unclear	Moderate

19	Küçük et al., 2025	No	Yes	Yes	Yes	No	Yes	Yes	Yes	Unclear	High
20	Okur Gümüşova et al., 2007	No	Unclear	No	Yes	No	Yes	Yes	Yes	Unclear	High
21	Özbek et al., 2024	No	No	No	Yes	No	Yes	Yes	Yes	Yes	High
22	Öner & Yeşilbağ, 2018	No	Yes	Yes	Yes	No	Yes	Yes	Yes	Yes	High
23	Paksoy et al., 2026	Yes	Yes	Yes	Yes	Yes	Yes	Yes	Yes	Unclear	Moderate
24	Şimşek et al., 2017	No	No	No	Yes	No	Yes	Yes	Yes	Yes	High
25	Tan et al., 2006	No	Unclear	Yes	Yes	No	Yes	Yes	Yes	Yes	High
26	Yavru et al., 2005	No	No	Yes	Yes	No	Yes	Yes	Yes	Yes	High
27	Yavru et al., 2013	No	Unclear	No	Yes	No	Yes	Yes	Yes	Unclear	High
28	Yazıcı et al., 2007	Unclear	Unclear	Yes	Yes	Unclear	Yes	Yes	Yes	Unclear	Moderate
29	Yazıcı et al., 2019	Yes	Yes	Yes	Yes	Yes	Yes	Yes	Yes	Unclear	Low
30	Yeşilbağ & Güngör, 2008	Yes	Yes	Yes	Yes	Yes	Yes	Yes	Yes	Unclear	Low
31	Yıldırım et al., 2009	Unclear	Unclear	Yes	Yes	Unclear	Yes	Yes	Yes	Unclear	Moderate
